# Intelligent Wearable Wrist Pulse Detection System Based on Piezoelectric Sensor Array

**DOI:** 10.3390/s23020835

**Published:** 2023-01-11

**Authors:** Yan-Yun Liu, Yu-Xiang Lv, Hai-Bin Xue

**Affiliations:** 1 Key Laboratory of Advanced Transducers and Intelligent Control System, Ministry of Education, Taiyuan University of Technology, Taiyuan 030024, China; 2 College of Physics, Taiyuan University of Technology, Taiyuan 030024, China

**Keywords:** the new piezoelectric sensor array, wearable wristbands, key points of radial artery, the pulse signal waveform, the slippery pulse and the normal pulse, human health status

## Abstract

The human radial artery pulse carries a rich array of biomedical information. Accurate detection of pulse signal waveform and the identification of the corresponding pulse condition are helpful in understanding the health status of the human body. In the process of pulse detection, there are some problems, such as inaccurate location of radial artery key points, poor signal noise reduction effect and low accuracy of pulse recognition. In this system, the pulse signal waveform is collected by the main control circuit and the new piezoelectric sensor array combined with the wearable wristband, creating the hardware circuit. The key points of radial artery are located by an adaptive pulse finding algorithm. The pulse signal is denoised by wavelet transform, iterative sliding window and prediction reconstruction algorithm. The slippery pulse and the normal pulse are recognized by feature extraction and classification algorithm, so as to analyze the health status of the human body. The system has accurate pulse positioning, good noise reduction effect, and the accuracy of intelligent analysis is up to 98.4%, which can meet the needs of family health care.

## 1. Introduction

The heart shoots blood into the artery and transmits it to the whole body through periodic and regular contraction and relaxation. After the blood is exchanged with tissues, it returns to the heart from the veins, forming a complete blood circulation cycle; in medicine, we call this the circulatory system. The pulse waveform formed by blood impact on the radial artery wall has periodic characteristics and carries biomedical information [1]. Because the radial artery of the wrist of the upper limb has the advantages of being close to the body surface, convenient to measure, and less affected by external factors, traditional Chinese medicine (TCM) looks there for Cun, Guan, and Chi; according to the academic language of pulse, “Take three parts of pulse, and each part is one cun” [2]. Namely, there are three key points of the radial artery: the radial protruding point is Guan, one cun above the Guan pulse is Cun, and one cun below the Guan pulse is Chi. Different forces are applied at key points to make the pulse in three states of floating, middle and sinking [3], and the amplitude, frequency and shape of the pulse signal are sensed, so as to discover, dig and extract the pulse condition information hidden behind the pulse signal [4]. According to the internal relationship between human health state and pulse condition, the human health state is analyzed to achieve the purpose of disease screening [5,6,7]. Although this method can quickly, safely and non-invasively realize the patient’s health detection and disease diagnosis, it is greatly affected by subjective factors and can find it difficult to accurately identify the pulse condition, which affects the analysis of human health status.

With the development of sensor technology and the rise of artificial intelligence, modern medicine tends to analyze human health status by collecting pulse signal waveforms at the radial artery through miniaturized and intelligent precision instruments [8,9,10,11,12,13]. In 1860, the Frenchman Etienne Jules Marey developed the pulse tracer [14], which depicted the pulse signal at the radial artery by tracing points through the mechanical principle. However, the pulse tracer was bulky, difficult to use accurately, and difficult for patients to accept. With the development of electronic technology, a variety of pulse signal detection systems with high accuracy have been designed. For example, Chen C, Sun Y et al. [15,16,17] used the principle that the voltages at the two ends of the resistors in the bridge were different under different pressures to package multiple piezoresistive sensors with the same type and size through special materials, and then the wires were bonded and electrically connected to the PCB board to form a sensor array, and a special material coating was added to form protection. Signals were collected at various positions on the radial artery axis at the same time. However, the classification of pulse waveforms is not carried out by machine learning algorithms. Chen Y. Y el et al. [18] used the principle of photoelectric reflection method to attach the photoelectric sensor to the radial artery on the surface of human skin to obtain the radial artery’s signal. However, most studies use optical sensors without pressurized structures, which makes it impossible to simulate the measurement method of traditional Chinese medicine to obtain pulse signals. Liu S et al. [19] detected the pulse signal at the radial artery through the hardware circuit design of the flexible circuit board combined with the tactile sensor attached to the wrist, which prevented the reverse pulse signal waveform to a certain extent. However, the research on covering the key points of the radial artery through the arrangement of the sensor array requires further intelligent positioning of the Cun, Guan and Chi. Lee Y et al. [20] placed an ultrasound probe at the human radial artery and achieved the acquisition of pulse signals by Doppler ultrasound technology. However, it is necessary to manually mark the positions of Cun, Guan and Chi obtained from the pulse diagnosis of traditional Chinese medicine, and measure the ultrasonic wave and pulse wave with a thin line wrapped around the marked measurement position. The current research [10,21,22,23] mainly realizes the detection of pulse signal waveform at the radial artery through the development of the piezoelectric sensor, but it is difficult for the polyvinylidene fluoride (PVDF) piezoelectric sensor to maintain consistency when the force is small, and the charge disappears faster under static force. The collected pulse signal has noise, and the pulse signal needs to be further denoised by software to make the pulse condition recognition more accurate.

Because the voltage at both ends of the new piezoelectric sensor is positively correlated with the pressure on its surface, it has the characteristics of strong anti-interference ability, high sensitivity, wide pressure range, good repeatability and strong consistency. In the software, the adaptive pulse-finding algorithm can realize the millimeter-level location of the radial artery key points, the wavelet transform, iterative sliding window, and predictive reconstruction algorithm can effectively eliminate the hardware circuit noise and external interference; the feature extraction and classification algorithm can then accurately identify the slippery pulse and the normal pulse, according to the principle that the slippery pulse corresponds to the pregnant women or the human body in the state of anemia, rheumatism, acute infection and fever, while the normal pulse corresponds to the human body in the state of good nutrition and good health [5,6,7], so as to achieve the purpose of disease screening. Therefore, in this paper, the pulse signal waveform was collected by the hardware circuit design that the main control circuit placed on the outside of the wrist band and the new piezoelectric sensor array combined with the flexible circuit board placed on the inside of the wrist band. The key points of the radial artery were located, the signal noise was reduced, and the slippery pulse and the normal pulse were identified to realize the intelligent analysis of human health status by the above software algorithm.

## 2. System Structure and Design

### 2.1. System Constitutes

**Figure 1:**
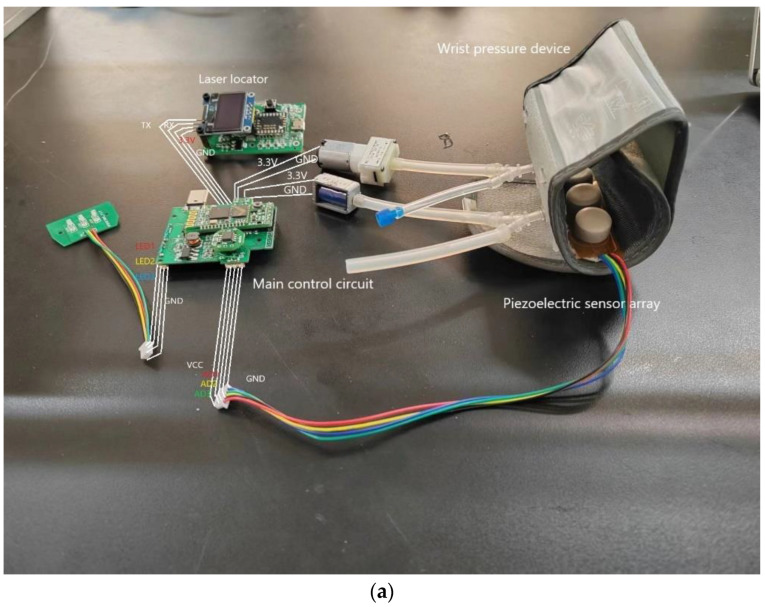

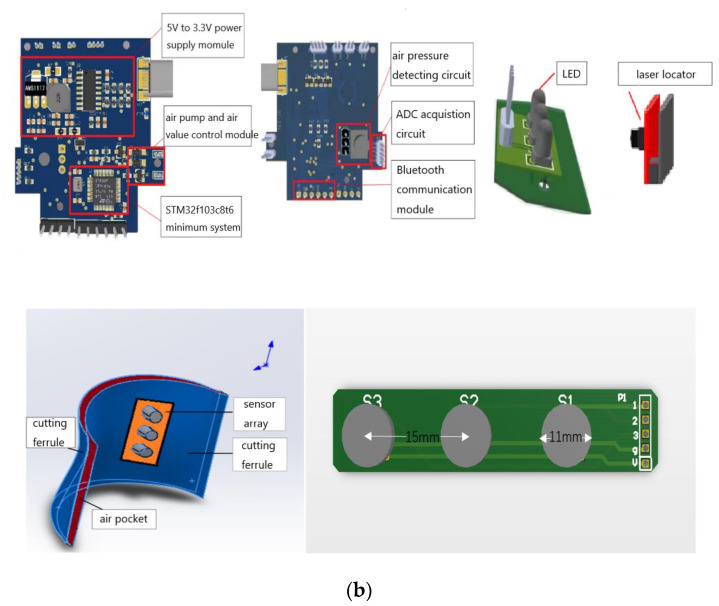
System constituents: (**a**) physical hardware; and (**b**) description of circuits.

The hardware of this system is composed of the main control circuit, the new piezoelectric sensor array, and the wrist pressure device, as shown in Figure 1. The main control circuit of the system takes STM32F103C8T6 chip as the core, including four 12-bit AD acquisition circuits, XGZP6847A air pressure sensor, SC300IPN micro air pump and EVS-HD-3003 solenoid valve composed of air pressure detection and pressure circuit pressure detection and pressure circuits, laser positioning module composed of an ADNS-9800 chip, power management circuit composed of FM3209 chip, indicator circuit, HC-05 Bluetooth communication circuit, which realizes analog to digital signal conversion, wrist band pressure control, sensor displacement measurement, and Bluetooth data communication. The wrist pressure device is composed of an inflatable wristband, an air bag, radial stem bone ferrules, air filling and discharging ports, and corresponding hoses, which has the characteristics of small volume, fitting wrist skin and good air tightness. The new piezoelectric sensor array is composed of three 11 mm diameter cylindrical silica gel packaged new piezoelectric sensors arranged at an interval of 15 mm and integrated on a flexible circuit board, it can simulate the person’s index finger, middle finger and ring finger in the close state, which can effectively cover the corresponding position of Cun, Guan and Chi, so as to realize the acquisition and amplification of weak pulse signal.

### 2.2. New Piezoelectricity Sensor

**Figure 2:**
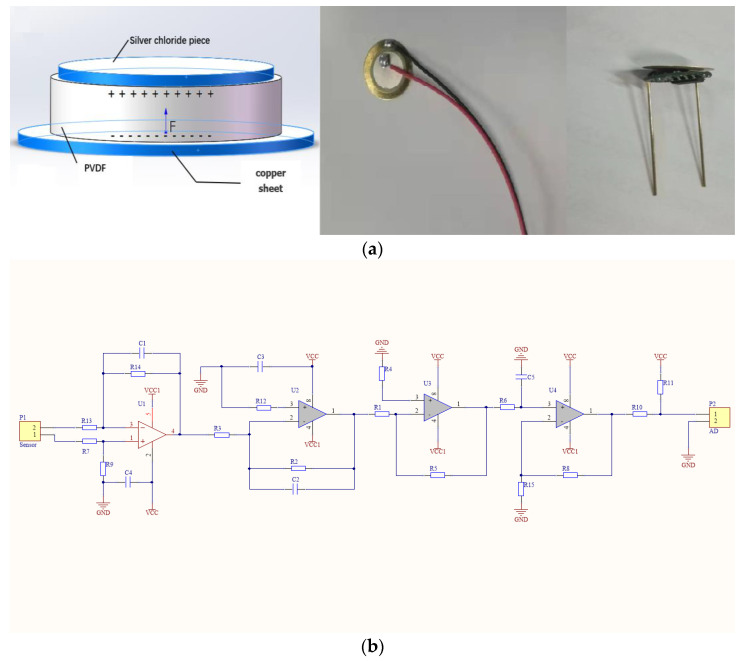

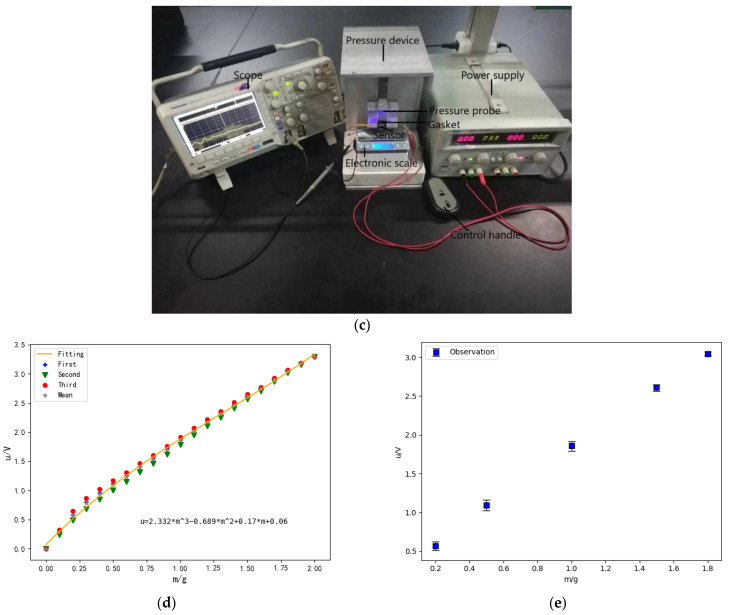
The working principle of the new piezoelectric sensor: (**a**) composite material; (**b**) sensor internal circuits; (**c**) experiment device; (**d**) piezoelectric effect; and (**e**) error analysis.

The new piezoelectric sensor is composed of copper sheet, piezoelectric ceramics (PZT), silver chloride sheet combined with differential charge amplifier circuit, voltage amplifier circuit and filter circuit. When the inner metal surface of the new piezoelectric sensor is under pressure, the piezoelectric ceramics (PZT) between the copper sheet and the silver chloride sheet is deformed. Due to the piezoelectric effect, the surface of the material forms a large number of ordered and isotropic charges, and the two ends of the metal are equivalent to a charged capacitor. After the U1 differential charge amplifier circuit, the U2 and U3 voltage amplifier circuits, and the U4 filter circuit, the composite material can identify 0 pC to 1 pC level charge transformation (corresponding to pressures in the range of 0 g to 2 g) and output with 0 V to 3.3 V working voltage. The experimental setup consists of MW MPS-3003C DC source, Tektronix MSO 2012 oscilloscope, LUILEC DC electric pushrod motor, motor operation handle, pressure probe, gasket, KFS-A electronic scale, and the new piezoelectric sensor. The constant speed motor push rod is controlled by the handle, and the pressure is applied to the sensor under the gasket by the pressure probe, and then the pressure is read by the electronic scale, and the oscilloscope shows the waveform. After that, the handle controls the constant speed motor push rod reset. The experiment is repeated to obtain the relationship between the pressure and the sensor output voltage. After the pressure test in the range of 0 g to 2 g, the output voltage of the sensor is positively correlated with the force of the piezoelectric material. When the external force disappears, the material recovers its original shape, the isotropic charge on the material surface disappears, and the sensor output voltage is zero. The piezoelectric effect of the new piezoelectric sensor is shown as follows:

(1) $$u=\frac{K}{1+j\omega R_{6}C_{5}}\frac{Q}{C_{1}}$$

(2) $$u=2.333m^{3}-0.689m^{2}+0.171m+0.068$$

where K is a constant, Q is the amount of charge, $C_{1}$ is the feedback capacitance, u is the output voltage, and m represents the external pressure. The frequency response range of the sensor is 0.5 Hz to 100 Hz, that is, the noise below 0.5 Hz and the high-frequency noise above 100 Hz are filtered through the circuit design, and the effective information of the pulse signal is retained to the maximum extent. The pressure response range is less than 0.206 kPa, that is, the sensor force range is 0 g to 2 g, the sensor surface area is $0.3025\pi \times 10^{-4}\;m^{2}$, the gravity acceleration g= 9.8 $m/s^{2}$, according to the pressure calculation formula  P=F/S=mg/S≤0.206 kPa. The sensitivity is 16.019 mV/Pa, that is, when the sensor range is 3.3 V, the maximum pressure is 0.206 kPa, so the sensor sensitivity is 3300 mV/206Pa=16.019 mV/Pa. The error is less than 5%. The sensor has the characteristics of strong anti-interference ability, high sensitivity, wide pressure range, good repeatability and strong consistency, which can be used for accurate detection of pulse signal waveform. The working principle of the new piezoelectric sensor is shown in Figure 2:

### 2.3. System Acquisition Process

Firstly, the system scanned the fixed position of the wrist through the new piezoelectric sensor array and the laser positioning module, collected three pulse signal data through three ADC at the sampling frequency of 125 Hz, obtained the waveform amplitude of the pulse signal and uploaded it through Bluetooth, combined with the adaptive pulse finding algorithm for data processing, positioned the three points with the largest average amplitude of the pulse waveform as the key points of the radial artery, and the new piezoelectric sensor array was deployed to the corresponding position by moving the wrist band.

Secondly, the system applied a continuous decreasing dynamic pressure of 4 mmHg/s to the wristband in the range of 60–160 mmHg, three pulse signals and one wristband pressure values were collected by four-channel ADC at a sampling frequency of 125 Hz; the data was uploaded to the computer for noise reduction by Bluetooth, and the waveform amplitude of pulse signal at different key points of radial artery with dynamic pressure was obtained so as to obtain the optimal pulse pressure P and the waveform amplitude of the corresponding pulse signal y $=u_{max}4096/3.3$.

**Figure 3:**
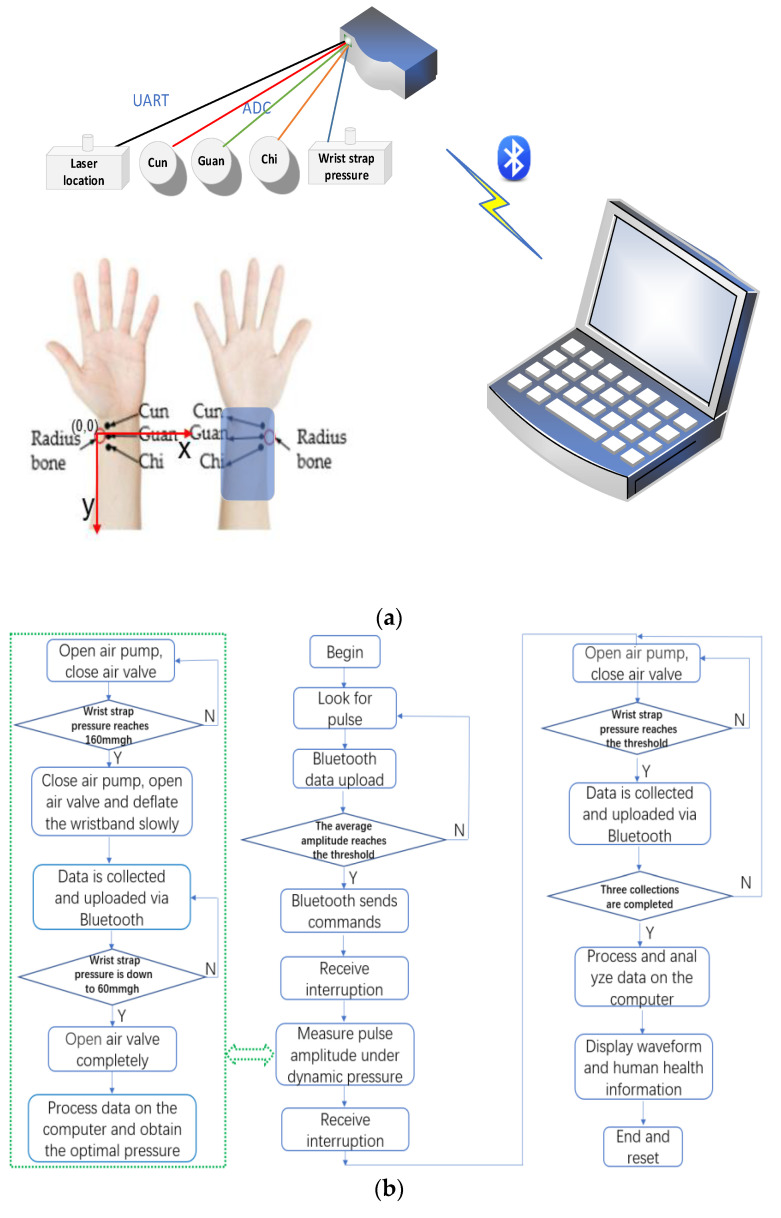

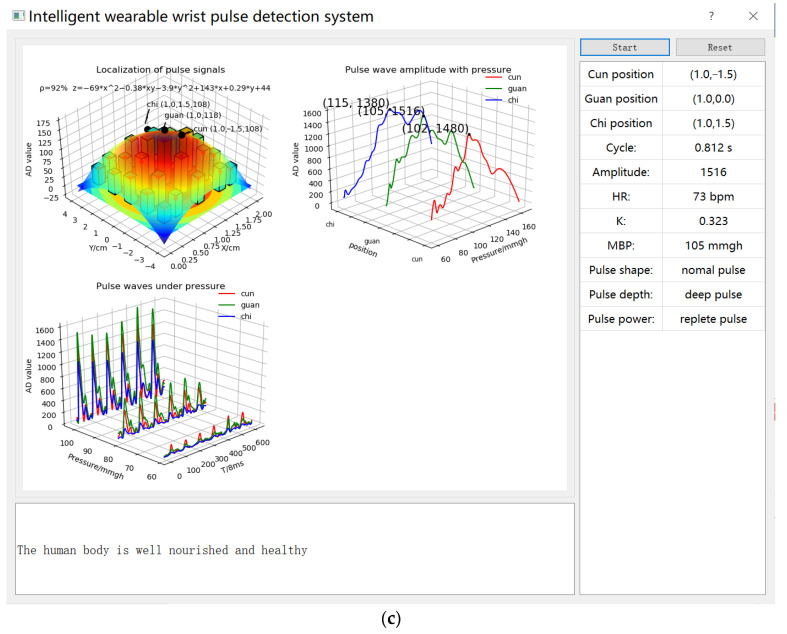
System acquisition process: (**a**) working principle of the system; (**b**) flow chart; and (**c**) system interface.

Finally, the system pressured the wrist band to 60 mmHg, 80 mmHg, 100 mmHg through the air pump, three pulse signal data and one wristband pressure value were collected by four-channel ADC with a sampling frequency of 125 Hz similarly, the data were uploaded to the computer by Bluetooth for noise reduction, feature extraction and analysis. Thus, the pulse signal waveform under three static air pressure, the recognition results of the slippery pulse and the normal pulse, and the analysis of human health state were obtained. The system acquisition process is shown in Figure 3:

## 3. Experimental Results

### 3.1. Adaptive Pulse Finding

**Figure 4:**
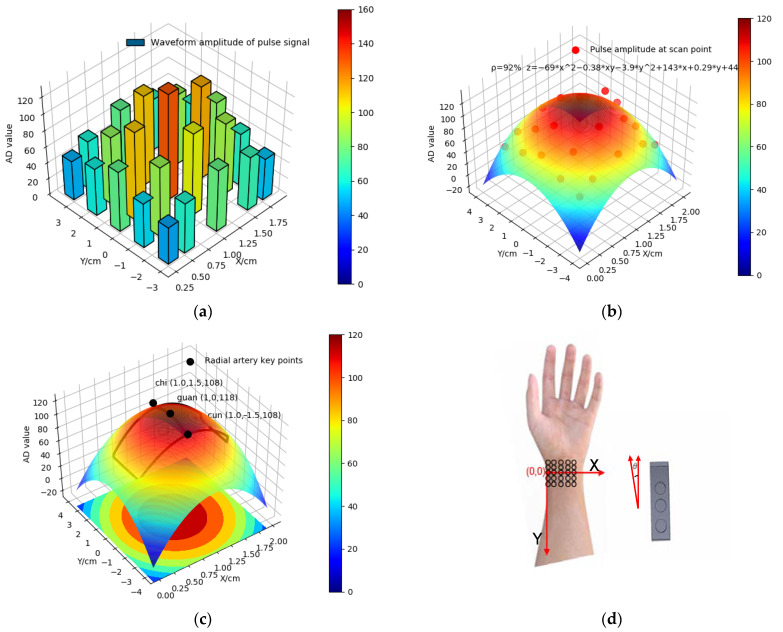
The principle of adaptive pulse finding. (**a**) Scan of fixed positions of the wrist; (**b**) the relationship between amplitudes and positions of the pulse signal; (**c**) radial artery key points location; and (**d**) deployment of the sensor array.

The key points of the radial artery are different in different people. Direct positioning of the key points of the radial artery by mechanical devices leads to large positioning errors, which affects the subsequent acquisition of pulse signal waveform and is not conducive to the analysis of human health status. In this system, the protrusion of the radial stem bone is first located as the origin, and the coordinates are established with the junction between the bottom of the palm and the arm as the x-axis, the arm direction as the y-axis, and the waveform amplitude of the pulse signal as the z-axis. The laser module is placed on the outside of the wrist band, and its laser head and lens are close to the arm to locate the new piezoelectric sensor and obtain the displacement of the new piezoelectric sensor. Then, the new piezoelectric sensor array, combined with the laser positioning module, is used to scan the fixed position of the wrist from top to bottom and from left to right along the x axis and y axis directions, as shown in Formula (3), a ternary data set (x,y,z) consisting of the position of the scanning point and the waveform amplitude of the pulse signal can be obtained. Then, the ternary data set is used for surface fitting and regression analysis to obtain Formula (4), and the relationship between pulse signal waveform amplitude and scanning point position is obtained. Then, conditional extreme value extraction is carried out (the distance between the three sensors is fixed at 1.5 cm), and the three points with the maximum average amplitude of pulse waveform $\left(x_{0},\;y_{0}\right)$, $\left(x_{1},\;y_{1}\right)$, $\left(x_{2},\;y_{2}\right)$ are obtained as the key points of the radial artery, as shown in Formula (5). Finally, by moving the wristband, the middle sensor is deployed to the position of the Guan pulse, and then the sensors at both ends are deployed to the position of the Cun pulse and the Chi pulse by adjusting the angle θ between the sensor array and the Y-axis, as shown in Formula (6), so as to realize the millimeter level positioning of the key points of the radial artery and the accurate deployment of the sensor array.

(3) $$\left(x,y\right)=\left[\begin{array}{ccccc} \left(0.25,-3\right) & \left(0.5,-3\right) & \left(1,-3\right) & \left(1.5,-3\right) & \left(1.75,-3\right)\\ \left(0.25,-1.5\right) & \left(0.5,-1.5\right) & \left(1,-1.5\right) & \left(1.5,-1.5\right) & \left(1.75,-1.5\right)\\ \left(0.25,0\right) & \left(0.5,0\right) & \left(1,0\right) & \left(1.5,0\right) & \left(1.75,0\right)\\ \left(0.25,1.5\right) & \left(0.5,1.5\right) & \left(1,1.5\right) & \left(1.5,1.5\right) & \left(1.75,1.5\right)\\ \left(0.25,3\right) & \left(0.5,3\right) & \left(1,3\right) & \left(1.5,3\right) & \left(1.75,3\right) \end{array}\right]\;$$

(4) $$z=a\left[0\right]x^{2}+a\left[1\right]xy+a\left[2\right]y^{2}+a\left[3\right]x+a\left[4\right]y+a\left[5\right]\;$$

(5) $$\{\begin{array}{l} {\left(x_{i\neq 1}-x_{1}\right)}^{2}+{\left(y_{i\neq 1}-y_{1}\right)}^{2}=2.25,\;\;i=0,1,2\\ \left(x_{1}-x_{0}\right)/\left(x_{2}-x_{1}\right)=\left(y_{1}-y_{0}\right)/\left(y_{2}-y_{1}\right)=1,\;\;y_{0}<y_{1}<y_{2}\\ f_{\left(x_{i},y_{i}\right)}=max\left(\frac{1}{3}\sum z_{i}\right),\;\;z_{i}\geq 60 \end{array}\;$$

(6) $$\theta =arctan\left(\left(x_{2}-x_{1}\right)/\left(y_{2}-y_{1}\right)\right)\;$$

where (x,y) is the coordinates of the scanning point, a[i] is the parameter, z is the waveform amplitude of the pulse signal, $f_{\left(x_{i},y_{i}\right)}$ is the maximum average amplitude, $\left(x_{i},y_{i}\right)$ are the coordinates of Cun, Guan and Chi, $z_{i}$ is the waveform amplitude of pulse signal at Cun, Guan and Chi, and θ is the angle between the sensor array and the Y-axis. The principle of adaptive pulse finding is shown in Figure 4:

### 3.2. Denoise Processing

The acquisition of pulse signals is usually affected by noise disturbances from hardware circuits and the baseline drift caused by self-respiration and body displacement. The short-time Fourier transform (STFT) of the signal can reflect the frequency characteristics of the signal well, the pulse signal with fixed frequency is selected through the window function to filter the noise, as shown in Formula (7), but its time resolution is fixed, and the details of the non-stationary signal can’t be extracted. The mean filtering method can filter out high-frequency noise, but there are extreme values and the phase angle offset. The baseline can be removed by cubic spline interpolation of the signal, but the stationarity of the signal edge becomes worse. In this system, the collected pulse data are decomposed by wavelet to obtain the scaling function coefficient CA5[n] and the wavelet function coefficient CD5[n], CD4[n], CD3[n], CD2[n], CD1[n], as shown in Formula (8). Then the wavelet function coefficient is denoised by the soft and hard threshold compromise method, as shown in Formula (9). Then the scaling function coefficient and the wavelet function coefficient are reconstructed by wavelet to obtain the smooth pulse signal waveform. Finally, the iterative sliding window algorithm is used to remove the phase angle offset by setting the sliding window width as the pulse signal period and using the coordinate rotation formula as the window function recursion, as shown in Formula (10), remove the baseline, as shown in Formula (11), and the complete pulse signal waveform with smooth and steady edge is obtained.

(7) $$X\left[M,K\right]=\overset{+\infty }{\underset{n=-\infty }{\sum }}x\left[n\right]w\left[n-k\right]e^{-j\frac{2\pi mn}{N}}\;$$

(8) $$X\left(t\right)=\underset{n}{\sum }CA\dot{x}\left[n\right]\varphi_{\dot{x},n}\left(t\right)+\overset{\infty }{\underset{x=\dot{x}}{\sum }}\underset{n}{\sum }CDx\left[n\right]\psi_{x,n}\left(t\right)\;$$

(9) $$CDx\left[n\right]=\{\begin{array}{c} sgn\left(CDx\left[n\right]\right)\left(\left|CDx\left[n\right]\right|-\lambda n\right),\left|CDx\left[n\right]\right|\geq N\\ \;\;\;\;\;\;\;\;\;\;\;\;\;\;\;\;\;0,\;\;\;\;\;\;\;\;\;\;\;\;\;\;\;\;\;\;\;\;\;\;\;\;\;\;\;\;\;\;\;\;\;\;\;\;\left|CDx\left[n\right]\right|<N \end{array},\;\;\;\;\;\;\;\;\;\;\;\;\;\;\;\;\;\;\;\;\;\;\;\;\;\;\;\;\;\;\;\;\;\;N=1.43median\left(CD1\left[n\right]\right)\sqrt{2{log}_{e}500}$$

(10) $$\left[n,x\left(n\right)\right]=\left[n,f\left(n\right)\right]*\left[\begin{array}{cc} 1 & -tan\theta \\ 0 & 1 \end{array}\right],\;\;\left(-\pi /2\leq \theta \leq \pi /2\right)\;$$

(11) y(n)=x(n)−min(x(n)),  (0≤n≤T) 

**Figure 5:**
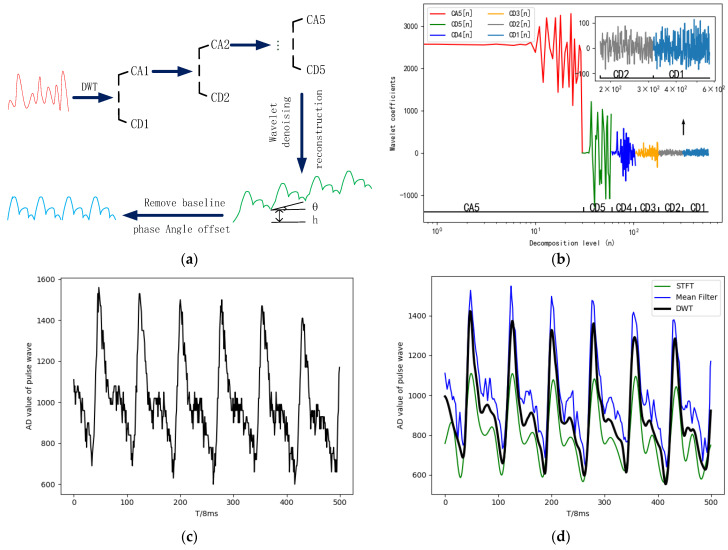

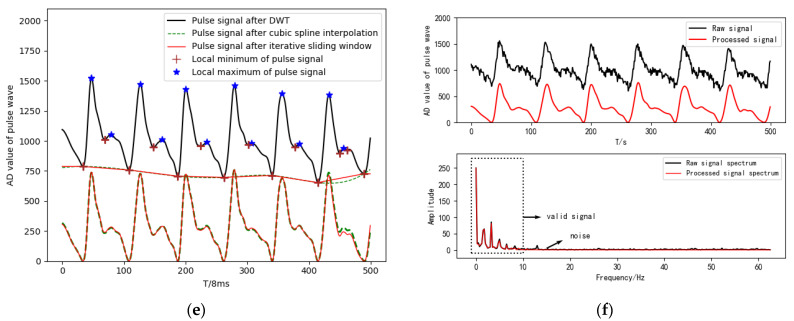
Wavelet transform and iterative sliding window processing of the pulse signal. (**a**) The pulse signal preprocessing flow; (**b**) Wavelet decomposition of the pulse signal; (**c**) Raw signal; (**d**) Hardware circuits noise removal; (**e**) The baseline and the phase angle offset removal; (**f**) Amplitude-frequency characteristics of the pulse signal.

**Figure 6:**
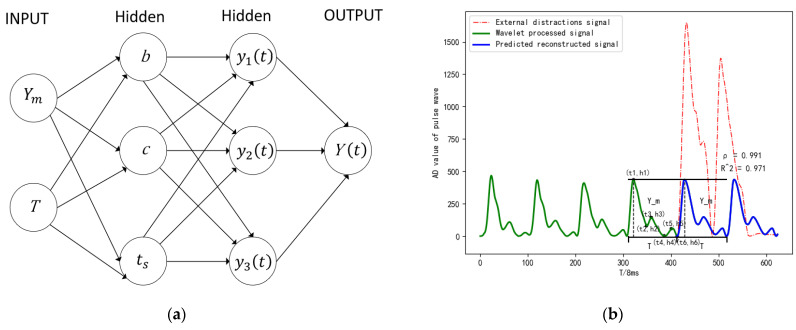

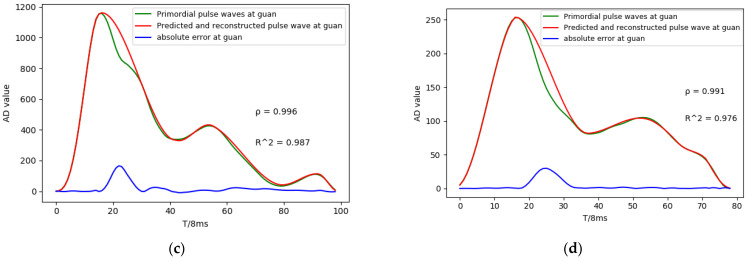
Prediction and reconstruction of the pulse signal: (**a**) neural network structure; (**b**) principles of prediction and reconstruction; (**c**) prediction and reconstruction effects of the normal pulse; and (**d**) prediction and reconstruction effects of the slippery pulse.

In Formula (7), x[n] and w[k] are the sampled signal and window function, X[M,K] is the short-time Fourier transform signal. In Formula (8), X(t) is the pulse signal before wavelet decomposition, CDx[n] is the wavelet function coefficient, $CA\dot{x}\left[n\right]$ is the scale function coefficient $\sum_{n}CA\dot{x}\left[n\right]\varphi_{\dot{x},n}\left(t\right)$ is the scale component,$\sum_{x=\dot{x}}^{\infty }\underset{n}{\sum }CDx\left[n\right]\psi_{x,n}\left(t\right)$ is the detail component. In Formula (9), N is the threshold and λ is a constant. In Formula (10), θ is the angle between the connecting line and the horizontal line of the starting point and the ending point of the single period pulse, f(n) is the pulse signal processed by discrete wavelet transform (DWT). In Formula (11), T is the period of pulse signal, x(n) is the singly periodic pulse signal with the phase angle offset removed, and y(n) is the singly periodic pulse signal with the baseline drift removed. The wavelet transform and iterative sliding window processing of pulse signal are shown in Figure 5:

After wavelet transform and iterative sliding window processing, the mixed waveform which contains external interference in the pulse signal and some waveform amplitude and period mutations is predicted and reconstructed according to the pulse waveform of the last period. The waveform reconstruction by curve fitting requires a large number of signal sampling points, and the algorithm complexity is high. The pulse waveform is a composite waveform formed by superposition of main wave, tide wave and dicrotic wave according to frequency and modal reconstruction. Firstly, the main wave is analyzed by regression analysis according to the peak $Y_{m}$ and the period T, as shown in Formula (12), combined with the corresponding curvature of the main wave, as shown in Formula (13), and the waveform of the main wave is obtained, as shown in Formula (14). Then, the waveform of the tidal wave can be obtained by regression analysis based on peak $Y_{m1}$ and period $\left(T_{1}-b\right)$, as shown in Formula (15). Then, the waveform of the dicrotic wave can be obtained by regression analysis based on peak $Y_{m2}$ and period $\left(T-T_{1}\right)$, as shown in Formula (16). Then, the spatial and temporal domain relationships of the predicted main wave, the predicted tidal wave, and the predicted dicrotic wave are obtained in turn by the height of the peak and the trough of the main wave, tidal wave and dicrotic wave of the pulse signal of the last period h1,h2,h3,h4,h5,h6, and the occupied period t1,t2,t3,t4,t5,t6, as shown in Formula (17). Finally, the predicted main wave, the predicted tidal wave and the predicted dicrotic wave are superimposed in the period T and deployed to the corresponding position, as shown in Formula (18), and the predicted and reconstructed pulse signal waveform is obtained.

(12) $$f_{1}\left(t\right)=\{\begin{array}{ll} Y_{m}\left(3t_{s}t^{2}-2t^{3}\right)/t_{s}{}^{3} & ,\;\;0\leq t<t_{s}\\ Y_{m}\left(3(t_{s}-T){\left(T-t\right)}^{2}-2{\left(T-t\right)}^{3}\right)/{\left(t_{s}-T\right)}^{3} & ,\;\;t_{s}\leq t<T \end{array}\;$$

(13) $$k_{1}\left(t\right)=\{\begin{array}{ll} 1 & ,\;\;0\leq t<t_{s}\\ \left(1-c\right)\left(b-t\right)/\left(b-t_{s}\right)+c & ,\;\;t_{s}\leq t<b\\ ce^{-{\left(\left(t-b\right)/\left(T-b\right)\right)}^{3}} & ,\;\;b\leq t<T \end{array}\;\;\;\;\;\;\;\;\;\;\;\;\;\;\;\;\;\;\;\;\;\;\;\;\;\;\;\;\;\;\;\;\;\;\;\;\;\;\;\;\;\;\;\;\;\;\;\;$$

(14) $$y_{1}\left(t\right)=k_{1}\left(t\right)f_{1}\left(t\right),\;\;0\leq t<T\;$$

(15) $$y_{2}\left(t\right)=\{\begin{array}{ll} Y_{m_{1}}\left(3\left(t_{s_{1}}-b\right){\left(t-b\right)}^{2}-2{\left(t-b\right)}^{3}\right)/{\left(t_{s_{1}}-b\right)}^{3} & ,\;\;b\leq t<t_{s1}\\ Y_{m_{1}}\left(3\left(t_{s_{1}}-T_{1}\right){\left(T_{1}-t\right)}^{2}-2{\left(T_{1}-t\right)}^{3}\right)/{\left(t_{s_{1}}-T_{1}\right)}^{3} & ,\;\;t_{s1}\leq t<T_{1} \end{array}$$

(16) $$y_{3}\left(t\right)=\{\begin{array}{ll} Y_{m_{2}}\left(3\left(t_{s_{2}}-T_{1}\right){\left(t-T_{1}\right)}^{2}-2{\left(t-T_{1}\right)}^{3}\right)/{\left(t_{s_{2}}-T_{1}\right)}^{3} & ,\;\;T_{1}\leq t<t_{s2}\\ Y_{m_{2}}\left(3\left(t_{s_{2}}-T\right){\left(T-t\right)}^{2}-2{\left(T-t\right)}^{3}\right)/{\left(t_{s_{2}}-T\right)}^{3} & ,\;\;t_{s2}\leq t<T \end{array}$$

(17) $$\{\begin{array}{l} Y_{m}=h1\\ Y_{m_{1}}=h3-y_{1}\left(t3-\left(t6-T\right)\right)\\ Y_{m_{2}}=h5-y_{1}\left(t5-\left(t6-T\right)\right)\\ t_{s_{1}}=t3-\left(t6-T\right)\\ t_{s_{2}}=t5-\left(t6-T\right)\\ T_{1}=t4-\left(t6-T\right)\\ b=t2-\left(t6-T\right)\\ t_{s}=t1-\left(t6-T\right) \end{array}$$

(18) $$Y\left(t6+t\right)=y_{1}\left(t\right)+y_{2}\left(t\right)+y_{3}\left(t\right),\;\;0\leq t<T$$

where $T_{1}$ is the time from starting point to the tidal wave valley, b is the time from starting point to the main wave valley, $t_{s}$ is the time occupied by the ascending branch, $t_{s_{1}}$ is the time from starting point to the tidal wave peak, $t_{s_{2}}$ is the time from starting point to the dicrotic wave peak, $k_{1}\left(t\right)$ is the corresponding curvature of the signal, $f_{1}\left(t\right)$ is the fundamental signal. *c* is a constant, $y_{1}\left(t\right)$ is the predicted main wave waveform, $y_{2}\left(t\right)$ is the predicted tidal wave waveform, $y_{3}\left(t\right)$ is the predicted dicrotic wave waveform, and Y(t) is the predicted reconstructed pulse signal waveform. The neural network is used to adjust b to change the width of the main wave, adjust $t_{s}$ to change the proportion of the rising branch, and adjust c to change the curvature of the waveform, so that the predicted reconstructed pulse waveform has high fitting degree, obvious characteristics, and meets the requirements of amplitude and period. The prediction and reconstruction of the pulse signal is shown in Figure 6:

### 3.3. Feature Extraction and Classification

**Figure 7:**
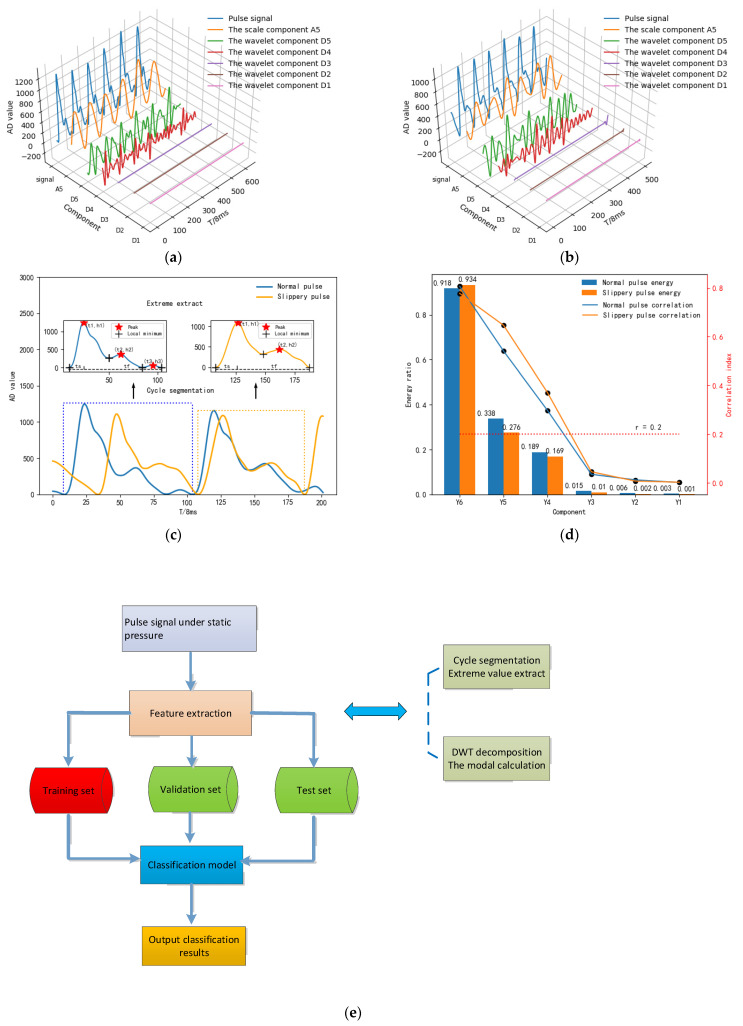

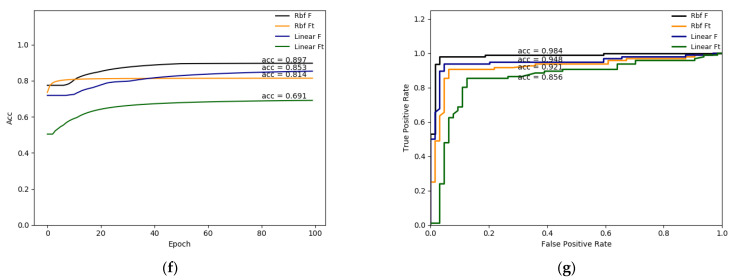
The principle of feature extraction and classification: (**a**) the normal pulse and its wavelet component; (**b**) the slippery pulse and its wavelet component; (**c**) extraction of the spatial-temporal features; (**d**) extraction of the modal energy features; (**e**) classification process of pulse signals; (**f**) classification performance of NN; (**g**) classification performance of SVM.

The difference in waveform between the sliding pulse and the normal pulse is the absence or presence of the dicrotic wave. Due to the influence of human factors, there is a condition in which the dicrotic wave are inconspicuous in the collected pulse signal waveform. The extreme value extraction method is used to determine whether the pulse signal waveform contains dicrotic wave, which leads to the inaccurate identification of the sliding pulse and the normal pulse, thus affecting the analysis of human health status. We commissioned Shanghai Bayes Health Technology Co., Ltd., Shanghai, China, to collect the pulse of 140 subjects from various TCM clinics through this system, including 100 healthy men and women aged 20–30 years, 30 men and women aged 20–30 years who were in the state of cold or fever, and 10 women aged 20–30 years who were in pregnancy. A total of 1260 sets of corresponding pulse signals were divided into the normal pulse and the slippery pulse labels. Then the pulse dataset was randomly shuffled and rearranged, of which 60% was used as the training set, 20% was used as the validation set, and 20% was used as the test set. Through the feature extraction and classification algorithm, this system first extracts the spatial-temporal features $F_{t}$ and the modal energy features $F_{y}$ of pulse signal, and then uses the machine learning method to train the classification model to identify the slip-pulse and normal pulse, so as to realize the accurate analysis of human health status. The principle of feature extraction and classification is shown in Figure 7:

Firstly, the system processes the signal by wavelet transform, iterative sliding window and prediction reconstruction algorithm. Then, the ratio of ascending and descending branches $t_{s}$/$t_{f}$, the height ratio of main wave, tidal wave and dicrotic wave $h_{1}:h_{2}:h_{3}$, and the occupied period ratio $t_{1}:t_{2}:t_{3}$ can be obtained by the period segmentation and the extreme value extraction, so as to obtain the spatial-temporal features $F_{t}\left\{t_{s}/t_{f},t_{1}:t_{2}:t_{3},h_{1}:h_{2}:h_{3}\right\}$, as shown in Table 1:

Then, by calculating the correlation coefficient $r=cov\left(Y,Y_{i}\right)/\sigma \sigma_{i}$ between the processed pulse signal Y and the components after wavelet decomposition *Y_i_*, the residual components $Y_{1}$, $Y_{2}$, $Y_{3}$ with r≤ 0.2, and the detail components $Y_{4}$, $Y_{5}$ and the scale component $Y_{6}$ with r > 0.2 are obtained. Extract wavelet components of r > 0.2 to calculate the corresponding mode energy $y_{i}=\left|\left|Y_{i}\right|\right|/\left|\left|Y\right|\right|$, so as to obtain the modal energy features $F_{y}\left\{y_{6},y_{5},y_{4}\right\}$, as shown in Table 2:

Finally, in the case of a small data set and three independent repeated experiments, the classification models of the slippery pulse and the normal pulse are trained, respectively, by neural network (NN) and support vector machine (SVM), linear kernel function (Linear) and radial basis function (Rbf), combined with spatial time domain characteristic $F_{t}$ and mixed characteristic $F\left\{F_{t},F_{y}\right\}$, and the recognition effects of different classification models are compared to select the best classification model, as shown in Table 3:

Since the slippery pulse corresponds to the pregnant women or the human body is in the state of anemia, rheumatism, acute infection and fever, while the normal pulse corresponds to the human body is in the state of good nutrition and health [5,6,7], the system uses support vector machine (SVM), radial basis function (Rbf), combined with mixed features to train the slippery pulse and the normal pulse classification model, identify the slippery pulse and the normal pulse, realize the intelligent analysis of human health status, and its accuracy is up to 98.4%.

## 4. Discussion

In this paper, an intelligent wearable wrist pulse detection system based on a new piezoelectric sensor array is designed. The system adopts the main control circuit, new piezoelectric sensor array, wearable wristband hardware circuit design, combined with the algorithm of adaptive pulse finding, wavelet transformation, iterative sliding window, prediction and reconstruction, feature extraction and classification to locate the key points of the radial artery, reproduce the pulse signal waveform under the air pressure of different wristbands, eliminate the hardware circuit noise and external interference, extract the pulse signal features and identify the slippery pulse and the normal pulse, so as to realize the intelligent analysis of human health status. The sensitivity of the new piezoelectric sensor in the system is 16.019 mV/Pa, the pressure response range is 0.206 kPa, and the error is less than 5%. The pulse finding algorithm can realize millimeter-level location of radial artery key points, and the pulse signal waveform with smooth waveform, stable edge and obvious waveform characteristics can be obtained by noise reduction processing. The accuracy rate of the slippery pulse and the normal pulse identified by feature extraction and classification algorithm is 98.4%, which can accurately identify human health status.

The proposed system and other pulse measurement devices presented in recent studies are summarized in Table 4. Clemente F [22] proposed a piezo-film-based measurement system. Jessica [9] proposed a wearable pulse-taking device, and Sun Y [16] proposed a wearable pulse wave monitoring system.

In conclusion, the wrist pulse acquisition system is feasible, and has the characteristics of small size, accurate pulse positioning, good noise reduction effect, and accurate intelligent analysis. It is suitable for different groups of people and meets the needs of family health care. In the future, we would integrate the accelerometer and gyroscope into the wearable system to effectively detect the arm’s motion state and eliminate the inhibitory motion artifacts. At the same time, more experiments will be carried out on the basis of traditional Chinese medicine to perfect the pulse database, and the relationship between pulse condition and human health status will be established through machine learning, and the patients with different diseases and healthy personnel can be classified and identified precisely, which is of great significance for the objective research of traditional Chinese medicine pulse diagnosis.

## 5. Patents

“A low-power pulse signal acquisition device” are filed by China Patents (2021-10-22, CN113520338A[P]).

## Figures and Tables

**Table 1:** The spatial-temporal features.

State	$t_{s}$ $/t_{f}$	$t_{1}:t_{2}:t_{3}$	$h_{1}:h_{2}:h_{3}$
Normal pulse	0.195	1:3.117:5.529	1:0.288:0.139
	0.171	1:3.643:6.429	1:0.252:0.114
	0.196	1:3.235:5.059	1:0.358:0.096
	0.176	1:3.354:6.118	1:0.375:0.129
	0.183	1:3.123:5.631	1:0.256:0.131
	0.186	1:3.508:5.991	1:0.259:0.108
Slippery pulse	0.262	1:2.813:0	1:0.345:0
	0.278	1:2.941:0	1:0.393:0
	0.246	1:1.171:0	1:0.363:0
	0.281	1:1.185:0	1:0.329:0
	0.274	1:1.026:0	1:0.372:0

**Table 2:** The modal energy features.

State	$y_{6}$	$y_{5}$	$y_{4}$
Normal pulse	0.918	0.325	0.198
	0.909	0.364	0.167
	0.914	0.344	0.189
	0.917	0.338	0.188
	0.883	0.393	0.228
	0.889	0.386	0.219
Slippery pulse	0.936	0.307	0.197
	0.928	0.286	0.181
	0.934	0.301	0.194
	0.927	0.279	0.173
	0.931	0.295	0.186

**Table 3:** Comparison of the effects of classification models.

Classification Model	Kernel Function	Major Class Features	Accuracy Rate
NN	Linear	$F_{t}$	0.691
		F	0.853
	Rbf	$F_{t}$	0.814
		F	0.897
SVM	Linear	$F_{t}$	0.856
		F	0.948
	Rbf	$F_{t}$	0.921
		F	0.984

**Table 4:** Comparisons with other pulse measurement systems.

System	Clemente F	Jessica	Sun Y	Proposed
Wearable wristbands	Yes	Yes	Yes	Yes
Type of sensors	Piezoelectricity	Piezoresistive	Piezoresistive	Piezoelectricity
Looking for pulse	No	No	No	Yes
Prediction and reconstruction	No	No	No	Yes
Intelligent classification	No	No	No	Yes
Year of publication	2010	2017	2018	2023

## Data Availability

Data will be available by email upon reasonable request.

## References

[1] Charlton P.H., Mariscal Harana J., Vennin S., Li Y., Chowienczyk P., Alastruey J. (2019). Modeling arterial pulse waves in healthy aging: A database for in silico evaluation of hemodynamics and pulse wave indexes. Am. J. Physiol.-Heart Circ. Physiol..

[2] Chu Y.W., Luo C.H., Chung Y.F., Hu C.S., Yeh C.C. (2014). Using an array sensor to determine differences in pulse diagnosis—Three positions and nine indicators. Eur. J. Integr. Med..

[3] Luo C.-H., Su C.J., Huang T.Y., Chung C.Y. (2016). Non-invasive holistic health measurements using pulse diagnosis: I. Validation by three-dimensional pulse mapping. Eur. J. Integr. Med..

[4] Wu H.K., Ko Y.S., Lin Y.S., Wu H.T., Tsai T.H., Chang H.H. (2017). The correlation between pulse diagnosis and constitution identification in traditional Chinese medicine. Complement. Ther. Med..

[5] Feng X., Feng L., Gao H., Wang Q.S., Xia Y.M., Xu Z.X., Wang Y.Q. (2022). Characteristics of Pulse Parameters in Patients with Polycystic Ovary Syndrome Varied at Different Body Mass Index Levels. Evid.-Based Complement. Altern. Med..

[6] Gan Z., Zhang D., Huang Z., Chen L. (2018). A preliminary study on discriminant analysis of syndrome types in the recovery period of stroke in traditional Chinese medicine. BioMed Res. Int..

[7] Lin F., Zhang J., Wang Z., Zhang X., Yao R., Li Y. (2021). Research on feature mining algorithm and disease diagnosis of pulse signal based on piezoelectric sensor. Inform. Med. Unlocked.

[8] Li J.Q., Li R., Chen Z.Z., Deng G.Q., Wang H., Mavromoustakis C.X., Song H., Ming Z. (2018). Design of a continuous blood pressure measurement system based on pulse wave and ECG signals. IEEE J. Transl. Eng. Health Med..

[9] Kabigting J.E., Chen A.D., Chang E.J.H., Lee W.N., Roberts R.C. (2017). Mems pressure sensor array wearable for traditional Chinese medicine pulse-taking. 2017 IEEE 14th International Conference on Wearable and Implantable Body Sensor Networks (BSN).

[10] Murphy J.C., Morrison K., McLaughlin J., Manoharan G., Adgey A.J. (2011). An Innovative Piezoelectric-Based Method for Measuring Pulse Wave Velocity in Patients with Hypertension. J. Clin. Hypertens..

[11] Khan M.U., Aziz S., Akram T., Amjad F., Iqtidar K., Nam Y., Khan M.A. (2021). Expert hypertension detection system featuring pulse plethysmograph signals and hybrid feature selection and reduction scheme. Sensors.

[12] Naqvi S.Z.H., Aziz S., Khan M.U., Asghar N., Rasool G. (2020). Emotion Recognition System using Pulse Plethysmograph. 2020 International Conference on Emerging Trends in Smart Technologies (ICETST).

[13] Yang P., Stankevicius D., Marozas V., Deng Z., Liu E., Lukosevicius A., Dong F., Xu L., Min G. (2016). Lifelogging data validation model for internet of things enabled personalized healthcare. IEEE Trans. Syst. Man Cybern. Syst..

[14] Roguin A. (2006). Scipione Riva-Rocci and the men behind the mercury sphygmomanometer. Int. J. Clin. Pract..

[15] Chen C., Li Z., Zhang Y., Zhang S., Hou J., Zhang H. (2019). A 3D Wrist Pulse Signal Acquisition System for Width Information of Pulse Wave. Sensors.

[16] Sun Y., Dong Y., Gao R., Chu Y., Zhang M., Qian X., Wang X. (2018). Wearable pulse wave monitoring system based on MEMS sensors. Micromachines.

[17] Wang P., Zuo W., Zhang H., Zhang D. (2012). Design and implementation of a multi-channel pulse signal acquisition system. Proceedings of the 2012 5th International Conference on BioMedical Engineering and Informatics.

[18] Chen Y.Y., Chang R.S., Jwo K.W., Hsu C.C., Tsao C.P. (2015). A non-contact pulse automatic positioning measurement system for traditional Chinese medicine. Sensors.

[19] Liu S., Zhang S., Zhang Y., Geng X., Zhang J., Zhang H. (2018). A novel flexible pressure sensor array for depth information of radial artery. Sens. Actuators A Phys..

[20] Lee Y.J., Lee J., Lee H.J., Kim J.Y. (2009). A study on correlation between BMI and oriental medical pulse diagnosis using ultrasonic wave. 13th International Conference on Biomedical Engineering.

[21] McLaughlin J., McNeill M., Braun B., McCormack P.D. (2003). Piezoelectric sensor determination of arterial pulse wave velocity. Physiol. Meas..

[22] Clemente F., Arpaia P., Cimmino P. (2010). A piezo-film-based measurement system for global haemodynamic assessment. Physiol. Meas..

[23] Chiu Y.Y., Lin W.Y., Wang H.Y., Huang S.B., Wu M.H. (2013). Development of a piezoelectric polyvinylidene fluoride (PVDF) polymer-based sensor patch for simultaneous heartbeat and respiration monitoring. Sens. Actuators A Phys..

